# Molecular Diagnosis and Identification of a Novel Pathogenic Variant in Autosomal Dominant Polycystic Kidney Disease (ADPKD): A Case in Full Bloom

**DOI:** 10.7759/cureus.97021

**Published:** 2025-11-16

**Authors:** Dimitrios Pallas, Achilleas Betsikos, Vasiliki Zafeirouli, Virginia Geladari, Nikolaos Sabanis

**Affiliations:** 1 Department of Hepatology, Avicenne Hospital, Paris, FRA; 2 Department of Internal Medicine, General Hospital of Trikala, Trikala, GRC; 3 Department of Nephrology, General Hospital of Trikala, Trikala, GRC

**Keywords:** autosomal dominant polycystic kidney, autosomal dominant polycystic kidney and liver disease, heterozygous pathogenic variant, intracranial aneurysm, polycystin-2

## Abstract

We report a case of a 58-year-old woman presenting with worsening arterial hypertension and a history of symptomatic stone disease. Detailed family pedigree analysis, appropriate imaging studies, and genomic sequencing identified a novel pathogenic variant in the PKD2 gene, c.1262C>A (p. Ala421Glu), confirming the diagnosis of autosomal dominant polycystic kidney disease (ADPKD). To the best of our knowledge, this case study represents the first documented report of the causative role of c.1262C>A (p. Ala421Glu) variant in the ADPKD phenotype.

## Introduction

Polycystic kidney diseases (PKDs) encompass a wide spectrum of inherited disorders with variable clinical features and causative genetic factors leading to progressive decline in renal function [1]. PKDs follow either the autosomal dominant or recessive Mendelian pattern of inheritance. Polycystin-1 (PC1) and polycystin-2 (PC2) proteins are encoded by PKD1 and PKD2 genes, located on chromosomes 16 and 4, respectively [2]. PC1 and PC2 form a receptor/ion channel protein complex on the primary cilia of renal tubular cells, which is crucial for renal cell differentiation during embryogenesis [3]. Pathogenic variants in PKD1 and PKD2 genes are the leading cause of autosomal dominant polycystic kidney disease (ADPKD), the most common type amongst PKDs, accounting for more than 90% of affected individuals [1,4].

ADPKD affects 1 in 400 to 1 in 1000 live births worldwide, making it the most common monogenic inherited cause of end-stage kidney disease (ESKD) [1,4]. ADPKD is a multisystemic disease presenting with a diverse range of clinical manifestations. However, the disease manifests predominantly with numerous bilateral renal cysts, primarily localized to the collecting duct system. Cyst formation and progression demonstrate significant inter-individual variability, influenced by genotypic differences, although the overall rate of cystic expansion typically follows an indolent course. Hypertension is the most common and usually the earliest symptom in patients with ADPKD [5].

Proteinuria, high urinary sodium excretion, nephrolithiasis, and urinary tract infections are also common clinical characteristics of renal involvement in ADPKD [5,6]. Glomerular filtration rate (GFR) displays a gradual decline at a rate of 4-5 mL/minute per year, usually after 50 or 70 years of age for PKD1 and PKD2 patients, respectively [7]. Intracystic hemorrhage represents a frequent complication resulting in hematuria, especially when communicating with the collecting duct system. Cyst rupture is commonly presented as acute flank pain, fever, and hemodynamic instability, suggesting retroperitoneal bleeding. Enlargement of the cysts can cause intrarenal urinary stasis, which is a major contributor to kidney stone formation, leading to recurrent episodes of renal colics, hematuria, and obstructive uropathy. Despite its nomenclature focusing on kidney disease, there is also a plethora of extrarenal manifestations. Polycystic liver disease (four or more cysts) coexists in more than half of the patients by the age of 60 [8]. Mitral valve prolapse, aortic stenosis, and other heart malformations have also been described. Although rare, a subarachnoid or intracerebral hemorrhage, caused by a ruptured intracranial aneurysm, is the most fatal extrarenal complication in ADPKD patients, occurring with higher prevalence in women [9].

Therefore, ADPKD is a multifaceted disease affecting diverse organs and presenting with a constellation of renal and extra-renal manifestations. Herein, we present a case of a 58-year-old female patient with a full-blown ADPKD phenotype due to a novel mutation in the PKD2 gene, identified through next-generation sequencing (NGS).

## Case presentation

A 58-year-old woman presented as an outpatient to the Nephrology Department of General Hospital of Trikala, Trikala, Greece, complaining of inadequate blood pressure control. On presentation, clinical examination was unremarkable. Her blood pressure was 146/90 mmHg, oxygen saturation was 97% in ambient air, and heart rate was 68 beats/min. Body temperature was 36.6°C.

Her past medical history included arterial hypertension dating back 15 years, dyslipidemia, and hyperuricemia. Recently, a comprehensive evaluation of the cardiovascular system was performed for the same reason, including an electrocardiogram, echocardiogram, and exercise stress test, as well as blood tests and urinalysis to check thyroid function, electrolyte issues, anemia, and renal function. No pathologic findings were reported apart from left ventricular hypertrophy compatible with the diagnosis of hypertensive heart disease. Prior hospitalizations included three episodes of renal colic accompanied by gross hematuria, attributable to renal stone disease over a period of 25 years. Her outpatient medical treatment consisted of a combination of valsartan/hydrochlorothiazide 160/12.5 mg once daily (o.d.), bisoprolol 5 mg o.d., allopurinol 150 mg o.d., and atorvastatin 20 mg o.d.

Her family history was also of note. Her mother underwent emergency nephrectomy due to spontaneous rupture of a renal cyst in the retroperitoneal space at the age of 85, while her sister was also hospitalized due to an episode of colic pain and gross hematuria without evaluation for a specific diagnosis.

Given the above-mentioned findings, a comprehensive diagnostic workup was initiated. As a first step, we performed a complete array of laboratory tests and urinalysis testing, assessing for microscopic hematuria. The results were unrevealing and can be seen in Table 1.

**Table 1:** Laboratory results.

Variables	Patient’s Values	Reference Range
Hemoglobin, Hematocrit (g/dL, %)	13.2, 39.8%	11.8-17.8, 37.9-47.9
White Blood Cells (x10^3^/μL)	8.52	4-10.8
Platelets (x10^3^/μL)	250	150-350
Blood Glucose (mg/dL)	92	75-115
Urea (mg/dL)	32	10-50
Creatinine (mg/dL)	0.73	0.40-1.10
Uric Acid (mg/dL)	6.4	2.3-6.1
Potassium (mmol/L)	4.35	3.5-5.1
Sodium (mmol/L)	140.0	136-145
Calcium (mg/dL)	9.5	8.1-10.4
Phosphorus (mg/dL)	3.8	2.6-4.5
Magnesium (mg/dL)	2.03	1.6-2.3
Serum Glutamic Oxaloacetic Transaminase (IU/L)	25	5-33
Serum Glutamic Pyruvic Transaminase (IU/L)	40	10-37
Alkaline Phosphatase (IU/L)	70	25-125
γ-Glutamic Transferase (IU/L)	28	9-35
Albumin (IU/dL)	4.38	3.4-4.8
Creatine Phosphokinase (mg/dL)	79	24-190
Total Cholesterol (mg/dL)	165	0-200
HDL-Cholesterol (mg/dL)	46	>45
LDL-Cholesterol (mg/dL)	95.4	0-155
Triglycerides (mg/dL)	118	0-150
Parathormone (pg/mL)	45.9	17-88
Thyroid Stimulating Hormone (TSH) (μIU/mL)	0.60	0.35-4.94
Ferritin (ng/mL)	137.75	10-204.5
1,25-(OH)_2_D_3_ (vitamin D)	18.7	7-53
Antineutrophil Cytoplasmic Antibodies (ANCA)	Negative	-
Antinuclear Antibodies (ANA)	Negative	-
Anti-Sjögren Syndrome A Antibodies (anti-SSA)	Negative	-
Anti-Sjögren Syndrome B Antibodies (anti-SSA)	Negative	-
Urinalysis - White Blood Cells (cells/high powered field)	3	0-5
Urinalysis - Red Blood Cells (cells/high powered field)	1	0-3

**Figure 1:**
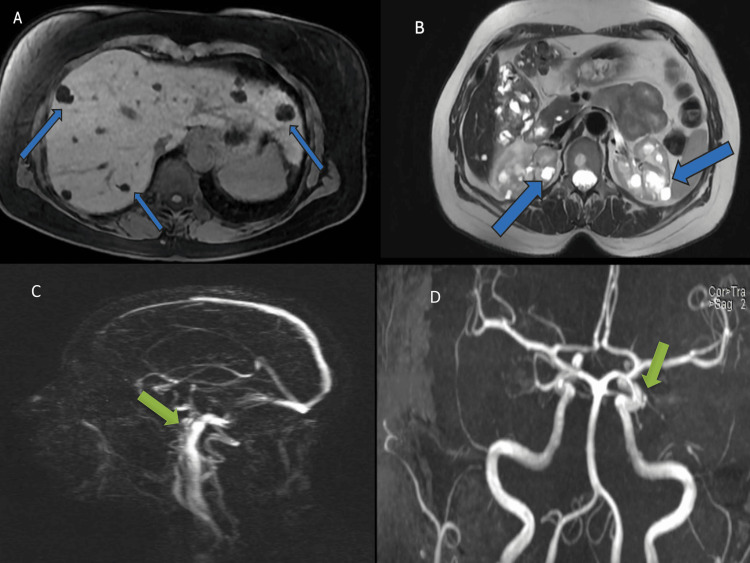
Abdominal magnetic resonance imaging (MRI) (A, B) and cerebral magnetic angiography (MRA) (C, D). Abdominal MRI revealed multiple bilobar hepatic cysts (A) and numerous bilateral sizeable renal cysts, some of them with hemorrhagic content, located predominantly in the renal cortex (B). Hepatic and renal cysts are pointed out with blue arrows. Cerebral MRA showed an aneurysm of the right internal carotid artery, in close proximity to the ophthalmic artery, measuring at 3.6 x 2.6 x 3.5 mm as pointed out with green arrows (C, D).

Upper abdominal and renal ultrasonography revealed multiple hepatic and bilateral renal cysts; the largest ones measured 2.5 cm in the kidneys and 5.6 cm in the liver. Further workup with an abdominal MRI was decided for better characterization, demonstrating bilobar hepatic cysts and confirming multiple bilateral renal cysts, some of which with hemorrhagic content (Figures 1A, 1B).

According to international guidelines from the Kidney Disease: Improving Global Outcomes (KDIGO), our patient met the criteria for the diagnosis of ADPKD [10]. Taking into consideration the female sex and the history of poorly controlled hypertension, we decided to obtain a magnetic resonance angiography (MRA) of the brain. Cerebral MRA revealed an aneurysm of the right internal carotid artery, proximal to the ophthalmic artery, measuring 3.6x 2.6x 3.5 mm (Figures 1C, 1D). This finding warranted strict hypertension control, which was achieved by adding lercadipin 10 mg twice daily to her treatment. Cardiology and neurosurgery consultations were obtained for a comprehensive approach and management of her situation. Recently, she underwent successfully an endovascular treatment with stent-assisted coil placement. Since then, she received dual antiplatelet therapy with clopidogrel 75 mg o.d and acetylsalicylic acid 100 mg o.d.

**Figure 2:**
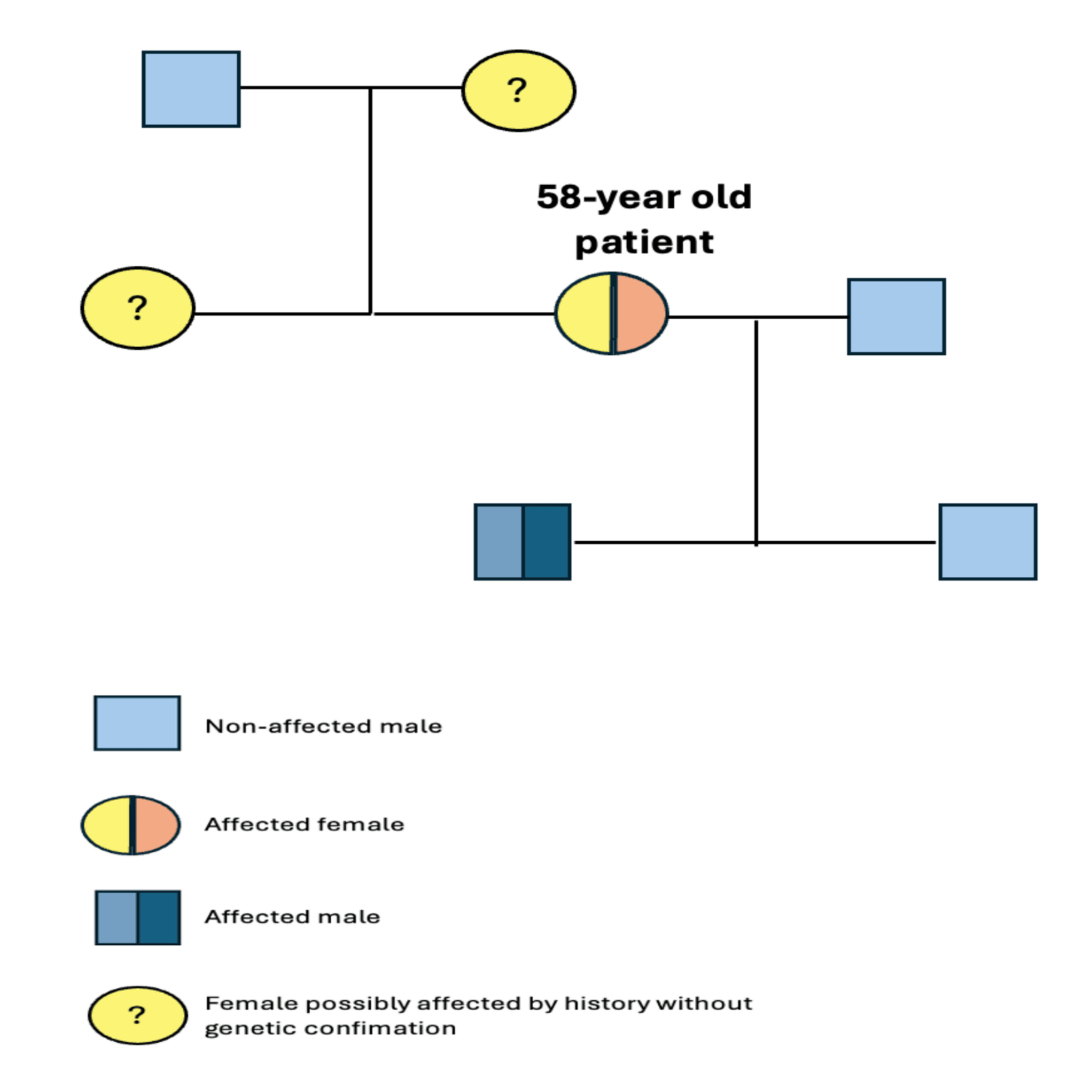
The pedigree chart of our patient's family.

As regards her sister’s vague medical history, broader family screening was initiated. The patient’s sister and one of her two sons displayed similar imaging features, as manifested by abdominal ultrasound results and magnetic resonance imaging findings. The family was counseled for genetic testing, and genomic sequencing in our patient was pursued. A heterozygous single-nucleotide variant (SNV) of the PKD2 gene was identified (c.1262C>A, p(Ala421Glu)). Her sister denied further workup, while sequencing of her 28-year-old son revealed the same pathogenic mutation. Certainly, all affected individuals were counseled to restrict sodium intake, maintain adequate hydration, and avoid certain medications, including anti-inflammatory drugs, beyond standard pharmacotherapy with angiotensin-converting enzyme inhibitors or angiotensin II receptor blockers. The patient’s three-generation family pedigree is shown in Figure 2.

## Discussion

ADPKD, once thought to affect exclusively the kidneys, is now an established multisystemic adult-onset genetic disease associated with major substantial morbidity. Although diagnostic tools and imaging modalities have largely evolved, ADPKD still remains quite underdiagnosed [11].

A comprehensive family history is the first step in the diagnostic approach to the disease. A history of ESKD, particularly at a young age, combined with polycystic appearance of the kidneys and hypertension, is usually sufficient for diagnosis. More often, a large kidney appearance with fluid-filled cysts is discovered incidentally when imaging is performed for unrelated indications. Genetic testing provides significant diagnostic and prognostic information in selected patients, although it is not required for establishing the diagnosis. Another crucial part of the diagnostic evaluation is the identification of high-risk patients for ESKD. The Mayo classification system for ADPKD is a validated prognostic tool that takes into consideration the patient’s age, height, and total kidney volume, which predicts a patient’s future estimated glomerular filtration rate (eGFR) decline [12].

ADPKD patients can benefit from specific treatment with tolvaptan, a selective V2 vasopressin receptor antagonist. These receptors are primarily expressed on the epithelial cells of the distal tubules, collecting tubules, and the collecting ducts, a major cyst formation site, directly regulating cyst growth [3].

In our patient, a normal eGFR warranted a more conservative approach, without the need for adding tolvaptan to her treatment. Optimal lifestyle and dietary changes, including a low-sodium diet, adequate water intake, weight management, avoidance of certain medications, primarily non-steroidal anti-inflammatory drugs, and strict blood pressure control, as well as regular monitoring, including renal ultrasonography, form the cornerstone of management in ADPKD patients with preserved renal function [10].

Interestingly, the novel mutation of the PKD2 gene highlights our patient’s phenotype. In this case, a cytosine to adenosine substitution at position 1262 within the coding DNA causes an alanine to glutamic acid substitution at position 421 of PC2 protein (c.1262C>A, p(Ala421Glu)). Initially, we used NCBI Genome Data Viewer to retrieve the wild-type coding DNA of the PKD2 gene (OMIM: 173910). Then, we searched for our mutation of interest, having only been reported once in the ClinVar genetic database, characterized as a missense variant of uncertain clinical significance (NM_000297.4(PKD2):c.1262C>A (p.Ala421Glu)).

PC2 belongs to the family of transient receptor potential proteins (TRPs), involved in calcium signaling pathways in primary cilia of the renal tubular cells [3]. Given its structure, PC2 can either form homomeric or heteromeric complexes with the PC1 protein, regulating cellular homeostasis.

**Figure 3:**
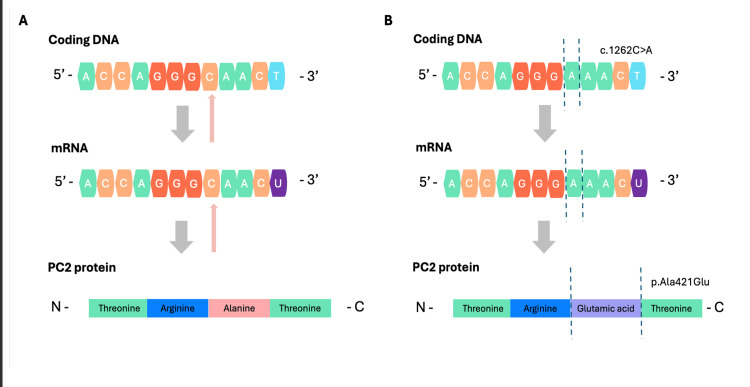
Normal (A) and mutated PKD2 gene (B) (NM_000297.4(PKD2):c.1262C>A), located in chromosome 4q21-22. (A) Wild-type coding DNA, mRNA, and PC2 protein. Small arrows indicate the site of interest (c.1262). (B) Mutation c.1262C>A (truncated lines) causes an adenine-to-cytosine substitution, subsequently altering the PC2 protein sequence by glutamic acid in place of alanine, as demonstrated by truncated lines (p. Ala421Glu). Credit: This image was created by Dimitrios Pallas, Achilleas Betsikos, and Nikolaos Sabanis through NCBI Genome Data Viewer.

The wild-type alanine at position 421 is a small, non-polar amino acid that is usually non-reactive based on standard biochemical properties; however, its substitution by glutamic acid (p.Ala421Glu), a large negatively charged amino acid with a longer methyl-side chain, introduces electrostatic properties that can hinder local folding (tertiary structure) and intermolecular communication properties of the PC2 protein. This hypothesis comes in accordance with the results of our in-silico analysis using the REVEL tool (Rare Exome Variant Ensemble Learner) that classified this variant as “likely pathogenic” [13]. The graphical representation of our analysis can be seen in Figure 3.

This nucleotide variation (rs1727652224) has previously been documented as a missense variant in clinical databases. However, to our knowledge, our report is the first to establish its pathogenic association with the ADPKD phenotype, supporting its classification as a disease-causing mutation.

## Conclusions

ADPKD is an inherited cause of ESKD with concomitant extrarenal complications, involving mutations of the PKD1 and PKD2 genes. Diagnostic accuracy lies in a combination of precise family history and imaging studies, while genome sequencing is limited to selected patients. In this article, we report a novel pathogenic variant of the PKD2 gene, highlighting the need for accurate genetic diagnosis and screening.
